# Iodine-doped carbon dots for fluorescence/computed tomography imaging and photodynamic therapy of tumor cells

**DOI:** 10.1039/d6ra00126b

**Published:** 2026-03-30

**Authors:** Binghan Guan, Jin Li, Tingting Liang, Yaoyao Zhang, Hanqin Wang, Bifu Hu, Xiaobo Wang

**Affiliations:** a Center for Translational Medicine, Suizhou Hospital, Hubei University of Medicine Suizhou 441300 People's Republic of China xbwang@hbmu.edu.cn; b Reproductive Medicine Center & Gynaecology Ward 2, Suizhou Hospital, Hubei University of Medicine Suizhou 441300 People's Republic of China; c Department of Radiology, Suizhou Hospital of Traditional Chinese Medicine Suizhou 441300 People's Republic of China 156689075@qq.com

## Abstract

Theranostic nanoagents have attracted considerable research interest due to their fascinating capability to simultaneously offer therapeutic and diagnostic functions. Here, novel iodine-doped carbon dots (I-CDs) were fabricated from arginine and iohexol *via* a facile hydrothermal strategy. I-CDs exhibited excellent stability and biocompatibility. Owing to a high quantum yield of 18.67%, the prepared I-CDs were suitable for the fluorescence imaging of tumor cells. Subcellular localization analysis showed that I-CDs were mostly distributed in lysosomes and mitochondria. Notably, the I-CD-treated A549 and HeLa cells simultaneously exhibited outstanding intracellular nitric oxide (NO) and reactive oxygen species (ROS) release capacities under an LED lamp illumination. The NO generation ability of I-CDs originated from the arginine moieties on their outer surface. The outcomes of *in vitro* investigations indicated that I-CDs were an effective nanophotosensitizer for the photodynamic therapy (PDT) of A549 and HeLa cells. Computed tomography (CT) scan data revealed that the Hounsfield unit (HU) value of I-CDs surpassed that of iohexol. Thereafter, the obtained I-CDs were successfully applied for performing the fast CT contrast imaging of mouse kidneys and bladders (within 1 minute); notably, I-CDs displayed negligible histopathological and hemolytic toxicity. I-CDs showed potential in the fields of fluorescence/CT bimodal imaging and PDT.

## Abstract 2

I-CDs for the fluorescence/CT imaging and photodynamic therapy of tumor cells.

## Graphical abstract

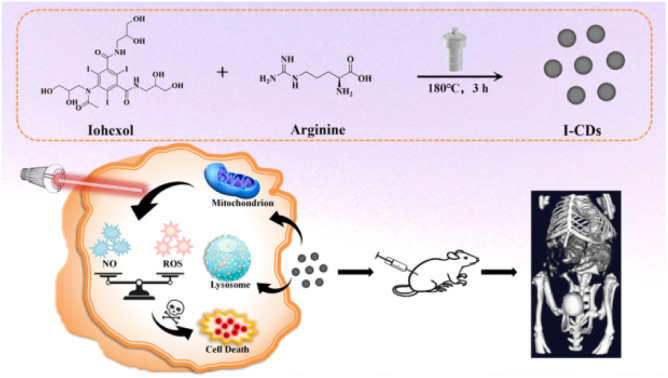

## 1. Introduction

As a universal diagnostic modality, computed tomography (CT) imaging is extensively utilized in clinical practice.^1,2^ The inherent drawback of CT imaging is its low contrast for soft tissues.^3^ Therefore, contrast agents are widely used for such tissues, which include the tissues of the intestinal tract, blood vessels, and tumors, during CT scans.^4^ At present, iodine-containing compounds are the most commonly employed CT contrast reagents due to their high mass attenuation coefficient.^1,5^ However, high concentrations of iodine contrast agents may cause adverse effects such as nausea, vomiting, shock, and renal function impairment.^6^ Other CT contrast agents such as gold-,^7–9^ bismuth-,^10,11^ gadolinium-,^12^ and lanthanide-containing^13^ nanomaterials have flourished in the field of theranostic medicine. The high cost of metal precursors and inherent toxicity of doped metal ions hinder their widespread usage in clinical practice.^14^

Carbon dots (CDs), as emerging materials with unique optical properties, have attracted tremendous attention in the realm of biomedicine.^15^ Bismuth- and gadolinium-based CDs have been used for fluorescence/CT/magnetic resonance (MR) multimodal imaging.^16^ Terbium-based CDs and hafnium-doped CDs have been successfully utilized in CT imaging research.^17,18^ Iodine-doped CDs have exhibited diverse applications in light-activated antibacterial activity, colitis therapy, and FL/CT bimodal imaging.^19–22^ Hence, it is an urgent necessity to develop novel iodine-based nanomaterials for the theranostic field.

Nitric oxide (NO), as a gaseous signaling molecule, participates in a variety of physiological and pathological processes, including vasodilation regulation, immune defense, inflammation modulation, and neural signal transmission.^23–26^ Photodynamic therapy (PDT) exhibits broad application prospects and promising clinical potential in the field of cancer treatment.^27,28^l-arginine, a natural basic amino acid, serves not only as an endogenous precursor of NO but also as a widely employed material in the preparation of functionalized CDs.^29^ These CDs have multiple biological applications such as cellular imaging, biochemical detection, gene delivery, antibacterial activity, and photodynamic therapy. Under irradiation by a laser or a light-emitting diode (LED), NO-releasing nanocomposites can induce DNA damage in tumor cells by inhibiting the activity of mitochondrial respiratory chain complexes, thereby leading to tumor cell apoptosis.^30^ Furthermore, the abundant reactive oxygen species (ROS) present in the tumor microenvironment can oxidize NO, aggravating oxidative stress injury, necrosis, or programmed apoptosis in tumor cells.^31,32^ Compared to PDT, which relies solely on ROS-mediated mechanisms, the synergistic strategy combining ROS and NO significantly enhances cytotoxicity against tumor cells and substantially improves therapeutic efficacy in cancer treatment.^33,34^

**Scheme 1:**
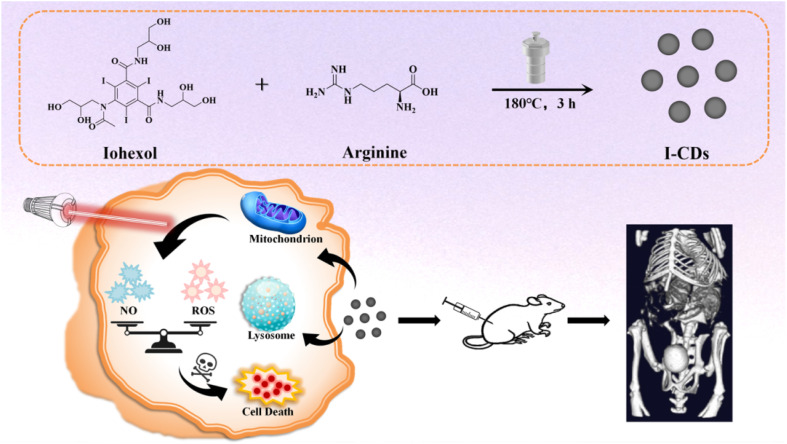
Schematic of the synthesis route of I-CDs and their utilization in fluorescence/CT imaging and photodynamic therapy of tumor cells.

Herein, a novel type of iodine-doped carbon dots was successfully fabricated from l-arginine and iohexol (Scheme 1). The as-prepared I-CDs displayed excellent optical stability and biocompatibility. Subcellular localization analysis showed that I-CDs were mostly distributed in lysosomes and mitochondria. Strikingly, the I-CD-treated A549 and HeLa cells simultaneously exhibited outstanding NO and ROS generation capacities under an LED lamp illumination. *In vitro* results demonstrated that I-CDs were an effective nanophotosensitizer for the photodynamic therapy (PDT) of A549 and HeLa cells. The obtained I-CDs were further applied for fast CT contrast imaging of mouse kidneys (within 1 minute), manifesting negligible histopathological and hemolytic toxicity. This study integrates the intrinsic X-ray-attenuation properties of iodine and the biocompatibility of carbon dots, achieving the dual-modal release of reactive oxygen species (ROS) and nitric oxide (NO) within a single nanosystem. Therefore, a multifunctional platform was established for bimodal fluorescence/CT imaging and photodynamic therapy.

## 2. Experimental

### 2.1. Materials

All chemicals and materials utilized in this study are carefully listed in the SI.

### 2.2. Ethics approval

Hemolysis assays were approved by the Ethics Committee of Suizhou Central Hospital, affiliated with the Hubei University of Medicine (ethics approval number: KY-2024-009-01). Animal experiments were approved by the Experimental Animal Ethics Review Committee of the Hubei University of Medicine (animal ethics approval number: Hubei University of Medicine Animal (Fu) no. 2024-Shi 131).

The blood samples used for the hemolysis assay were obtained from the Department of Reproductive Medicine, Suizhou Hospital, affiliated with the Hubei University of Medicine. Donors were healthy women with no history of infectious diseases such as tuberculosis, hepatitis B, or HIV/AIDS. Prior to blood collection, informed consent was obtained from the women or their family members, and the protocol was approved by the Ethics Committee of Suizhou Central Hospital, affiliated with the Hubei University of Medicine.

Six-week-old SPF-grade female BALB/c mice were provided by the Experimental Animal Center of the Hubei University of Medicine (license number: SYXK (Hubei) 2019-0031).

### 2.3. Methods

#### 2.3.1. Apparatus and characterization

The morphology of carbon dots was revealed *via* a high-resolution transmission electron microscope (HRTEM, FEI Tecnai G2 F20). The chemical structure and optical properties of carbon dots were determined using the following techniques: UV-visible spectrophotometry (Shimadzu, UV-2700), fluorescence spectroscopy (Horiba, FluoroMax-4), Fourier-transform infrared (FTIR) spectroscopy (Nicolet iS5), X-ray photoelectron spectroscopy (XPS, Thermo EscaLab 250Xi), and X-ray diffraction (XRD, Bruker D8 Advance). Live cell fluorescence photographs were captured using a fluorescence microscope (Olympus, IX73). Cell viability was measured using a microplate spectrophotometer (Biotek, Epoch). *In vitro* CT imaging was performed using a clinical CT imaging system (uCT960+, United Imaging Healthcare Co., Ltd, Shanghai, China). *In vivo* CT imaging was performed using a Quantum GX2/PerkinElmer Quantum GX2.

#### 2.3.2. Fabrication of I-CDs

Iohexol (0.1 g) and l-arginine (0.25 g) were solubilized in 10 mL of ultrapure water (UP water). The mixture was then stirred magnetically until a clear, transparent solution was obtained. The reaction mixture was poured into a 50 mL autoclave (a reinforced stainless-steel exterior and a specialized 50 mL polyphenylene polymer inner chamber) and hydrothermally treated at 180 °C for 3 hours. After cooling to ambient temperature, the reaction mixture was centrifuged at room temperature at 10 000 rpm for 10 minutes, and the *g*-force was 11 200 × *g*. Subsequently, the collected supernatant was passed through a hydrophilic polyethersulfone (PES) microporous filter membrane with a pore size of 0.22 µm. The filtered reaction solution was transferred to a dialysis membrane (MWCO = 500–1000 Da) and dialyzed against 2000 mL of ultrapure water for 24 hours, with the water renewed every 8 hours for a total of 3 cycles. The dialyzed sample was lyophilized to yield a light-yellow powder, which was kept at 4 °C in the refrigerator.

#### 2.3.3. Measurement of the photoluminescence quantum yield of I-CDs

Quinine sulfate was dissolved in a 0.1 M H_2_SO_4_ solution and employed as the reference (*Q*_R_ = 54%, *η* = 1.33). The quantum yield was derived according to the equation below:1
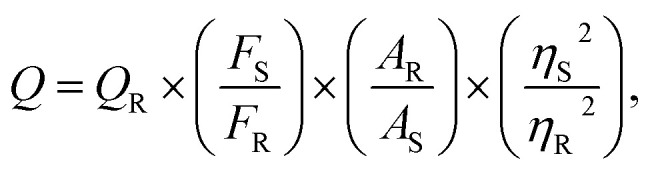
where the fluorescence quantum yield is denoted as *Q*. The roman letters (S and R) are used to distinguish between the sample and the reference standard, respectively. *F* denotes the fluorescence intensity that has been integrated. *A* represents the absorbance value of the sample measured at the excitation wavelength. *η* denotes the solvent's refractive index. To eliminate the optical reabsorption bias, the absorbance values of both carbon dots and quinine sulfate in a 10 mm optical-path quartz cuvette under the excitation wavelength were maintained below 0.1.

#### 2.3.4. Cell toxicity and haemocompatibility tests

Cell toxicity and haemocompatibility tests are carefully described in the SI.

#### 2.3.5. Cell imaging and subcellular localization of I-CDs

A549 and HeLa cells were seeded in 24-well plates at a density of 5 × 10^4^ cells per well and cultured for 24 hours at 37 °C with 5% CO_2_. The culture medium was replaced with a fresh medium containing I-CDs (600 µg mL^−1^) and incubated for another 4 hours. After washing three times with PBS, intracellular fluorescence imaging was performed using a fluorescence microscope.

For cellular uptake and subcellular localization analyses, cells in the logarithmic growth phase were digested, collected, reseeded in 24-well plates at 5 × 10^4^ cells per well, and cultured overnight. The medium was replaced with a serum-free medium containing 600 µg per mL I-CDs for the experimental group or a serum-free medium alone for the control group, followed by 4 h of incubation.

LysoTracker green and MitoTracker green working solutions were prepared at a ratio of 1 : 10 000 in a serum-free DMEM. After incubation with I-CDs, cells were washed with PBS, stained with Hoechst 33342 for 30 minutes, and then stained with LysoTracker green or MitoTracker green for 20 minutes. Fluorescence images were captured in blue, green, and red channels sequentially to avoid cross-channel interference. Co-localization was analyzed using ImageJ software to calculate the Pearson correlation coefficient (*r*), where −1 ≤ *r* ≤ 1. |*r*| > 0.5 indicates significant co-localization, while |*r*| < 0.3 indicates negligible co-localization.

#### 2.3.6. Cellular nitric oxide (NO) and reactive oxygen species (ROS) detection and calcein-AM/PI staining

For cellular nitric oxide (NO) and reactive oxygen species (ROS) detection, cell preparation steps in 24-well plates were the same as those for cytotoxicity assays. Then, I-CDs were added at a concentration of 600 µg mL^−1^ with 500 µL per well, followed by incubation in a cell incubator for 24 hours. The control group, control + LED group, I-CD group, and I-CD + LED group were set for light stimulation experiments, and the LED-treated groups were subjected to irradiation for 10 minutes (5 W cm^−2^, 400–800 nm). Subsequently, diluted DAF-FM DA (diluted at a 1 : 1000 ratio with the DAF-FM DA dilution buffer provided in the kit) or DCFH-DA (diluted at a 1 : 1000 ratio with the serum-free medium) probe was supplemented into the culture medium. After another 15 minutes of incubation, the cells were carefully rinsed thrice with PBS to remove the residual DAF-FM DA or DCFH-DA probe. Finally, the production of NO and ROS in A549 and HeLa cells was recorded and analyzed *via* an inverted fluorescence microscope.

Calcein-AM and PI were diluted at a ratio of 1 : 1 : 1000 with the dilution buffer provided in the kit. Cell preparation, I-CD treatment and light stimulation procedures were the same as described above. Subsequently, 300 µL of a calcein-AM/PI staining working solution was added to each well and incubated for 30 minutes. Cells were gently washed with PBS three times to remove free probes, and 500 µL of PBS was added to each well before imaging using an inverted fluorescence microscope.

#### 2.3.7. *In vivo* and *in vitro* CT imaging

I-CDs were prepared in PBS solutions with varying iodine concentrations and aliquoted into 1.5 mL Eppendorf tubes. The *in vitro* CT imaging and tomography of I-CD samples were performed using a clinical CT scanner, and the CT values of I-CD samples were compared with those of a clinical CT contrast agent (iohexol) at equivalent iodine concentrations. The following CT parameters were employed: slice thickness, 1.5 mm; slice interval, 0.75 mm; tube voltage, 90 kV; ad current intensity, 34 mA.

Six-week-old female BALB/c mice (*n* = 9) were randomly allocated to three groups for *in vivo* CT imaging. Mice were anesthetized by inhalation of 2% isoflurane, and their heads and limbs were fixed with adhesive tape. After 24 hours of fasting with no food, the mice were injected with I-CDs, iohexol, and PBS individually. Both I-CDs and iohexol were administered at a concentration of 9000 µg I per mL. CT scans were performed 1, 30 and 60 minutes after injection of I-CDs. After 48 hours, vital organs (heart, liver, spleen, lungs, and kidneys) were subjected to hematoxylin and eosin (H&E) coloration to assess the *in vivo* toxicological profile of I-CDs. The following CT parameters were employed: tube voltage, 70 kV; current intensity, 100 µA; and field of view: 86 mm.

#### 2.3.8. Hematoxylin and eosin (H&E) staining

After injection of the PBS buffer (or the iohexol solution or the I-CD solution) for 48 hours, the BALB/c mice were anaesthetized with isoflurane. The five major organs (heart, liver, spleen, lungs, and kidneys) of mice were collected. The organs were carefully rinsed with PBS buffer and fixed in 4% paraformaldehyde for 12–24 hours to prevent tissue autolysis. Subsequently, the organs were subjected to gradient dehydration: immersed in 70% ethanol for 4 hours, 80% ethanol for 2 hours, 95% ethanol for 2 hours, and 100% ethanol twice (30 minutes each time). The tissues were immersed in xylene twice (5–10 minutes each time) to ensure sufficient transparency for paraffin infiltration. The transparent tissues were embedded in melted paraffin, placed in an embedding machine for paraffin embedding and properly labeled. The embedded blocks were cooled in a −80 °C refrigerator until fully solidified and then trimmed. The trimmed blocks were cut into 4 µm-thick sections using a rotary microtome, mounted on glass slides, and dried in a 60 °C oven for 10 minutes.

Hematoxylin and eosin (H&E) staining was performed for the dried tissue sections. Firstly, tissue sections were deparaffinized with xylene twice (5 minutes each time) and then rehydrated through a graded ethanol series (100%, 95%, 80%, 70%, 3 minutes each). The sections were further stained with hematoxylin for 1 minute, rinsed with tap water, differentiated in 1% hydrochloric alcohol for several seconds, and washed again. Then, the sections were stained with eosin for 10 minutes and rinsed with water for 15 seconds. After dehydration through graded ethanol (70%, 80%, 95%, 100%, 3 minutes each) and clearing with xylene twice (5 minutes each), neutral balsam was added dropwise. Lastly, coverslips were placed carefully to avoid bubbles.

Pathological characteristics such as morphological changes in the major organs were observed in detail under an optical microscope to evaluate whether I-CDs could induce pathological damage to the main organs.

### 2.4. Statistical analysis

All measurements in this work were conducted three times. The data were processed statistically in GraphPad Prism 10.0 and ImageJ. Intergroup differences were evaluated by a two-tailed Student's *t*-test. *P*-value < 0.05 was deemed statistically notable. The levels of significance were denoted as follows: * indicated *P* < 0.05, ** indicated *P* < 0.01, and *** indicated *P* < 0.001.

## 3. Results and discussion

### 3.1. Fabrication of I-CDs

**Fig. 1:**
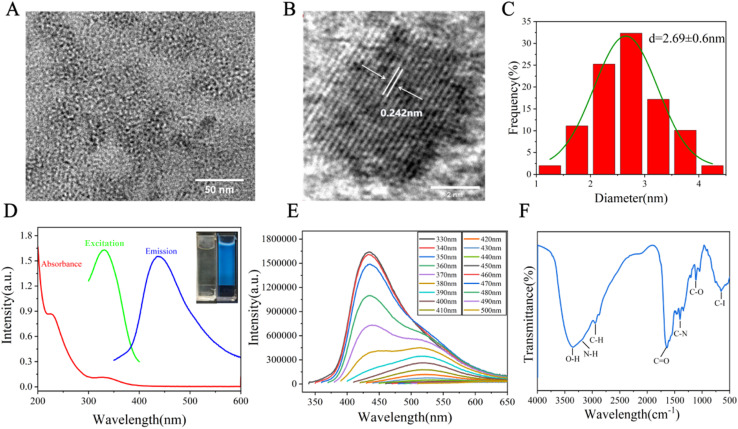
(A) TEM micrograph of I-CDs. (B) HRTEM image of I-CDs. (C) Particle size distribution of I-CDs. (D) UV-vis absorption, excitation and emission spectra of I-CDs. Inset: digital images of the I-CD aqueous medium in daylight (left) and under a 365 nm UV excitation (right). (E) Excitation-tunable fluorescence emission behavior of I-CDs. (F) FTIR profile of I-CDs.

I-CDs were prepared *via* a facile one-step hydrothermal method involving doping arginine with iohexol, based on a previously reported method with some modifications.^21^ In this system, arginine served as the carbon source, and iohexol was used as the passivating agent. The proposed formation mechanism of I-CDs is illustrated in Scheme 1. I-CDs exhibited quasispherical nanoparticles with a uniform particle size (Fig. 1A). Their high-resolution lattice fringe spacing was 0.242 nm, corresponding to the (100) plane of graphitic carbon,^35^ indicating the formation of ordered graphitized nanodomains inside, which provides structural support for the stability of the material (Fig. 1B). The particle size distribution showed that the average particle size of 100 randomly selected nanoparticles was 2.94 ± 0.6 nm (Fig. 1C), confirming their uniform nanoscale size.

The aqueous solution of I-CDs had two characteristic absorption peaks at 227 nm and 330 nm, which were attributed to the π–π* electron transition of the aromatic sp^2^ carbon structure (C=C bond) and n–π* electron transition of the oxygen or nitrogen containing group, respectively (Fig. 1D), suggesting that it has a conjugated carbon framework.^19,36^ Under excitation at 330 nm, its maximum emission peak was located at 434 nm with a large Stokes shift of 104 nm, which can effectively reduce reabsorption and self-absorption artifacts in fluorescence measurements, decrease excitation light leakage, and improve the signal-to-noise ratio of imaging.^37,38^

As shown in Fig. S1, time-resolved fluorescence decay analysis revealed that their fluorescence decay followed a biexponential kinetic model, yielding two lifetime components (*τ*_1_ = 0.19 ns and *τ*_2_ = 2.18 ns) and an average fluorescence lifetime of *t*_ave_ = 0.53 ns, where the short-lived component (*τ*_1_) corresponds to the intrinsic fluorescence of the carbon core, and the long-lived component (*τ*_2_) originates from the material's surface defect states or charge-separated species-states that are more likely to participate in redox reactions,^39^ thereby significantly improving the electron transfer efficiency, increasing the ROS yield, prolonging the electron transfer time to molecular oxygen, and facilitating ROS generation. The generated ROS further oxidize the l-arginine moieties conjugated on the surface of I-CDs to trigger NO release, wherein ROS first disrupts the mitochondrial membrane potential to initiate cellular damage under LED irradiation, and subsequent NO generated *via* ROS-mediated l-arginine oxidation inhibits the mitochondrial respiratory chain function, further amplifying oxidative stress and exacerbating mitochondrial dysfunction.^40^

Visualization results showed that the aqueous solution of I-CDs was clear and colorless under natural light and emitted a bright blue fluorescence under irradiation with a 365 nm UV lamp (inset of Fig. 1D), exhibiting excellent fluorescence properties. The UV-vis spectra of iohexol, arginine, and I-CDs are shown and compared in Fig. S2. Iohexol exhibited an obvious absorption peak at approximately 250 nm, while arginine had an absorption near 200 nm. In contrast, the absorption peak of I-CDs at 330 nm was completely separated from those of the two precursors, which confirms that the signal originates from the carbon dots.^41^ As the excitation wavelength increased from 330 nm to 500 nm, the emission peak red-shifted from 434 nm to 550 nm, showing significant excitation-dependent photoluminescence behavior (Fig. 1E), which is related to the presence of multiple emission centers with various sizes/energies and abundant surface states on the surface of carbon dots.^42^

FTIR characterization indicated that the surface of I-CDs had abundant hydrophilic functional groups. The broad peak at 3354 cm^−1^ corresponded to the stretching vibrations of O–H and N–H bonds,^43^ the peak at 2944 cm^−1^ was attributed to the stretching vibration of sp^3^ C–H bonds,^44^ and the characteristic peaks at 1641 cm^−1^, 1406 cm^−1^, and 1111 cm^−1^ corresponded to the vibrations of C=O, C–N, and C–O bonds,^45^ respectively (Fig. 1F). In addition, the absorption peak at 658 cm^−1^ was consistent with the C–I stretching vibration in halogen-doped systems, confirming that iodine is successfully embedded into the carbon framework.^19,21^ The FTIR spectra of arginine, iohexol, and I-CDs were compared to elucidate the structural evolution during hydrothermal carbonization and iodine doping (Fig. S3). The broad band at 3200–3500 cm^−1^, corresponding to O–H and N–H stretching vibrations,^17^ was retained in I-CDs, indicating the preservation of hydrophilic groups essential for biocompatibility. The saturated C–H stretching peaks at 2946 and 2862 cm^−1^ in arginine merged into a single peak at 2928 cm^−1^ in I-CDs,^43^ suggesting the condensation and rearrangement of alkyl chains during carbonization. Notably, as shown in Fig. S3, the C=O/C=C stretching peak shifted from 1623 cm^−1^ (arginine) and 1630 cm^−1^ (iohexol) to 1641 cm^−1^ in I-CDs, indicating the formation of a more conjugated sp^2^ carbon framework.^19^ The appearance of new peaks at 609 and 772 cm^−1^, characteristic of C–I bending and stretching vibrations, respectively, confirmed the successful incorporation of iodine from iohexol into the carbon dots. Meanwhile, the fine peaks associated with free carboxyl groups in arginine disappeared, indicating their conversion into amide or ester linkages *via* dehydration condensation. These spectral changes demonstrate that I-CDs are formed through the cocarbonization of arginine and iohexol, where the nitrogen-rich backbone of arginine and the iodine-containing moieties of iohexol are covalently integrated into a single nanostructure, endowing I-CDs with both fluorescent and CT imaging capabilities.^46^

**Fig. 2:**
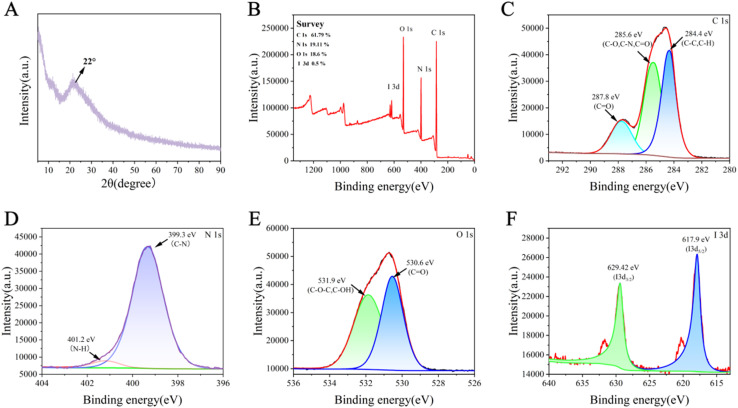
(A) XRD profile of I-CDs. (B) XPS survey spectrum of I-CDs. (C) C 1s, (D) N 1s, (E) O 1s, and (F) I 3d spectra of the prepared I-CDs.

The crystal structure of I-CDs was further verified by XRD patterns. As shown in Fig. 2A, a broad diffraction peak was observed at 2*θ* = 22°, corresponding to the (002) plane of amorphous or low-crystallinity carbon, indicating the existence of short-range ordered sp^2^-hybridized carbon layered structures inside I-CDs.^47^ According to Bragg's law, the corresponding (002) interplanar *d*-spacing was calculated to be 0.404 nm, which is larger than the interlayer spacing of ideal graphite (0.335 nm), suggesting the presence of short-range ordered graphite-like stacked structures with an expanded interlayer spacing in the material.^35,48^ The Debye–Scherrer formula was further used to analyze this peak (full width at half maximum, FWHM = 10°), and the average grain size along the stacking direction was calculated to be 0.81 nm, corresponding to a thickness of about 2–3 carbon layers.^49^ These results confirm that I-CDs have a composite structure, in which nanocrystals composed of very few graphite layers are embedded in an amorphous matrix, providing a structural basis for their excellent optical properties and stability.

XPS spectra were used to analyze the surface chemical structure and elemental distribution. The full XPS spectrum (Fig. 2B) exhibited four distinct signals at 284.97, 398.97, 530.97 and 618.97 eV, assigned to C 1s (61.79%), N 1s (19.11%), O 1s (18.6%) and I 3d (0.5%), respectively, confirming that I-CDs are mainly composed of carbon, nitrogen, oxygen and iodine. As shown in Fig. 2C, the C 1s spectrum was deconvoluted into three peaks at 284.4 eV (C–C/C–H), 285.6 eV (C–N/C–O) and 287.8 eV (C=O), indicating that the surface of I-CDs has abundant carboxyl, hydroxyl and nitrogen-containing groups.^24^ The N 1s spectrum (Fig. 2D) displayed two peaks at 399.3 eV and 401.2 eV, corresponding to pyridinic N/pyrrolic N (C–N) and graphitic N (C–N),^44^ respectively, indicating that nitrogen is successfully doped into the carbon skeleton in the form of heterocycles and forms C–N covalent bonds. The two characteristic peaks in the high-resolution O 1s spectrum (Fig. 2E) at 530.6 eV (C=O) and 531.9 eV (C–O–C/C–OH) further confirmed the abundance of oxygen-containing functional groups on the surface.^11^ Two characteristic peaks at 617.90 eV and 629.42 eV were observed in the high-resolution I 3d region (Fig. 2F), corresponding to I 3d_5/2_ and I 3d_3/2_, respectively, with an area ratio of approximately 3 : 2, which is consistent with spin–orbit splitting, proving that iodine is stably doped into the carbon skeleton in an oxidized state.^20,50^ These results directly confirm the successful doping of iodine into I-CDs.^21,24,50^

### 3.2. Stability of I-CDs

**Fig. 3:**
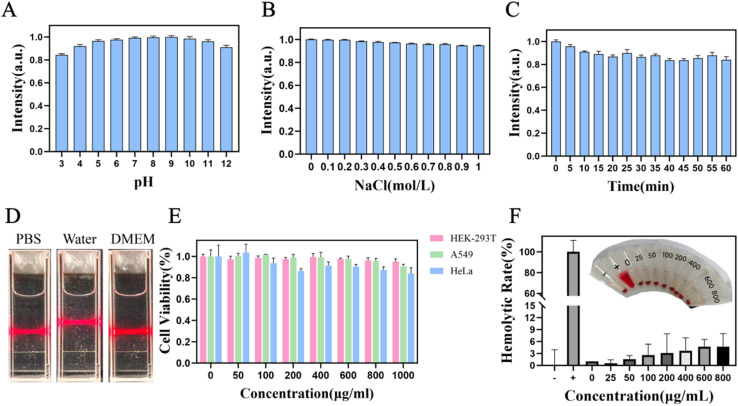
Fluorescence intensity of I-CDs at (A) different pH levels, (B) varying ionic strengths, and (C) varying UV irradiation times. (D) Tyndall effect of I-CD aqueous solutions. (E) Cell viability of HEK-293T, A549 and HeLa cells after 24 hour incubation with different concentrations of I-CDs. (F) Hemolytic activity of I-CDs at different concentrations.

We investigated the photoluminescence stability of I-CDs under various conditions (ionic environments, pH conditions, and UV exposure times). I-CDs maintained robust photoluminescence over a broad pH interval (pH 3–12, Fig. 3A). The emission intensity of I-CDs stayed nearly constant after supplementation with NaCl solutions at concentrations ranging from 0 to 1 mol L^−1^ (Fig. 3B). The I-CD aqueous solution was then exposed to UV light for 0–60 minutes. Even after 60 minutes of irradiation, the fluorescence intensity showed only a slight decrease (Fig. 3C). To evaluate the serum stability of I-CDs in the cell culture medium, the nanoparticles were dispersed in the complete cell culture medium supplemented with 10% fetal bovine serum (FBS), followed by incubation in a thermostatic shaker at 37 °C for 12 days under light-shielded conditions. The fluorescence intensity of I-CDs at an excitation wavelength of 334 nm was quantified *via* a fluorescence spectrophotometer, with column graphs constructed for the subsequent serum stability analysis. The results demonstrated that the relative fluorescence intensity of I-CDs remained above 85% over the 12 days incubation period, confirming their excellent stability in biological media (Fig. S4). An obvious Tyndall effect was observed in the I-CD aqueous solution (Fig. 3D), suggesting that the size of I-CDs in aqueous media was within the range of 1–100 nm.^51^ All these results confirmed that the as-prepared I-CDs had excellent fluorescence stability and favorable dispersity in water, suggesting their potential as a promising candidate for biological applications.

### 3.3. MTT and hemolysis assays of I-CDs

To assess the cellular toxicity of I-CDs, we utilized MTT assay to quantify the survival rate of HEK-293T, A549 and HeLa cells after treatment with I-CDs.^52^ HEK-293T cells showed a survival rate of 96.7% after treatment with I-CDs at a dose of 1000 µg mL^−1^ for 24 hours (Fig. 3E). Similarly, after treatment with I-CDs (1000 µg mL^−1^) for 24 hours, the cell viabilities of A549 and HeLa cells were 90.8% and 84.1%, respectively. Compared with the control group, the cell viabilities of the three cell lines under treatment with relatively high concentrations of I-CDs remained above 80% in all groups, with no statistically significant differences among groups (*P* > 0.05). These results indicate that I-CDs exhibit no obvious cytotoxicity, confirming their favorable biocompatibility.

Haemocompatibility was further assessed by the hemolysis assay. Human erythrocytes lysed in deionized water served as the positive control, human erythrocytes suspended in a PBS solution were used as the negative control, and a PBS solution was used as the blank. Test samples were prepared by suspending erythrocytes in PBS containing I-CDs at gradient concentrations. Even at a dose of 800 µg mL^−1^, the hemolysis ratio of I-CDs was approximately 4.7% (Fig. 3F), which is below the clinically acceptable threshold of 5%,^53^ confirming the excellent haemocompatibility of I-CDs. Moreover, the photographs of the hemolysis assay demonstrated that there was no obvious hemoglobin release or erythrocyte rupture in the red blood cell suspension. These results confirm that I-CDs possess excellent haemocompatibility and can effectively avoid hemolytic reactions caused by *in vivo* injection.

### 3.4. Cellular location behaviors of I-CDs

**Fig. 4:**
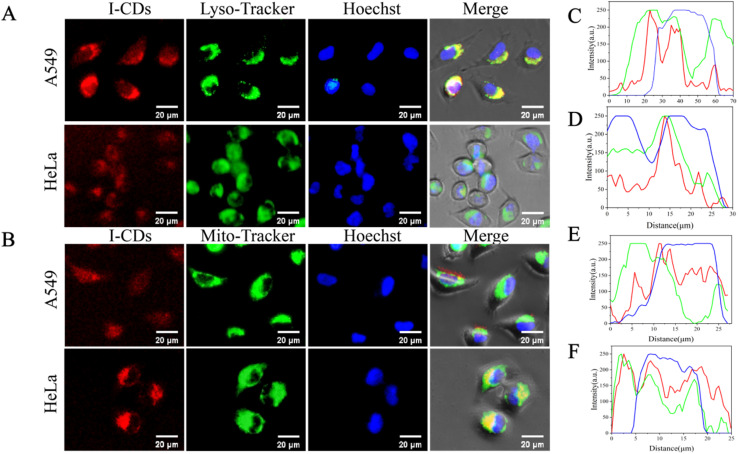
Fluorescent photographs of A549 cells incubated with I-CDs (600 µg mL^−1^) for 4 hours, followed by staining with (A) LysoTracker green and (B) MitoTracker green and Hoechst 33342. (C) Pearson's correlation coefficients analysed from I-CDs (red curve) and LysoTracker green (green curve) and Hoechst 33342 (blue curve) signs in A549 cells. (D) Pearson's correlation coefficients analysed from I-CDs (red curve) and LysoTracker green (green curve) and Hoechst 33342 (blue curve) signs in HeLa cells. (E) Pearson's correlation coefficients analysed from I-CDs (red curve) and MitoTracker green (green curve) and Hoechst 33342 (blue curve) signs in A549 cells. (F) Pearson's correlation coefficients analysed from I-CDs (red curve) and MitoTracker green (green curve) and Hoechst 33342 (blue curve) signs in HeLa cells. The scale bar represents 20 µm.

A549 and HeLa cells were exposed to I-CDs (600 µg mL^−1^) for 4 hours at 37 °C and 5% CO_2_. Under the red channel of an inverted fluorescence microscope, both types of cells displayed bright red fluorescence. To ascertain the suborganellar localization of I-CDs in tumor cells, the tumor cells treated with I-CDs were further labeled with lysosomal, mitochondrial and nuclear dyes, individually. Random linear regions of interest (ROIs) were generated on the merged images to measure Pearson's correlation coefficient *via* ImageJ software.^47,54^ As illustrated in Fig. 4, the red fluorescence signals emitted by I-CDs within the cells showed a high level of gemination with those of lysosomes and mitochondria probes. Pearson's correlation coefficients computed from I-CDs and LysoTracker green signals were 0.791 for A549 cells and 0.757 for HeLa cells. For mitochondria, Pearson's correlation coefficients determined from I-CDs and MitoTracker green signals were 0.796 for A549 cells and 0.886 for HeLa cells. For Hoechst 33342, Pearson's correlation coefficients determined from I-CDs and Hoechst 33342 signals were 0.489 for A549 cells and 0.511 for HeLa cells.^55,56^ An independent-sample *t*-test was used to compare the Pearson's correlation coefficients between A549 and HeLa cells. The Pearson's correlation coefficients for mitochondrial colocalization differed significantly between A549 and HeLa cells (*P* < 0.01). However, there was no significant difference in the Pearson's correlation coefficient for lysosomal and nuclear colocalization between A549 and HeLa cells (*P* > 0.05). These findings indicate that I-CDs can target lysosomes and mitochondria synchronously. These experimental results and data analysis demonstrate that after cellular internalization, I-CDs are simultaneously and efficiently localized in lysosomes and mitochondria, but barely in the cell nucleus.

### 3.5. Cellular NO and ROS generation of I-CDs

**Fig. 5:**
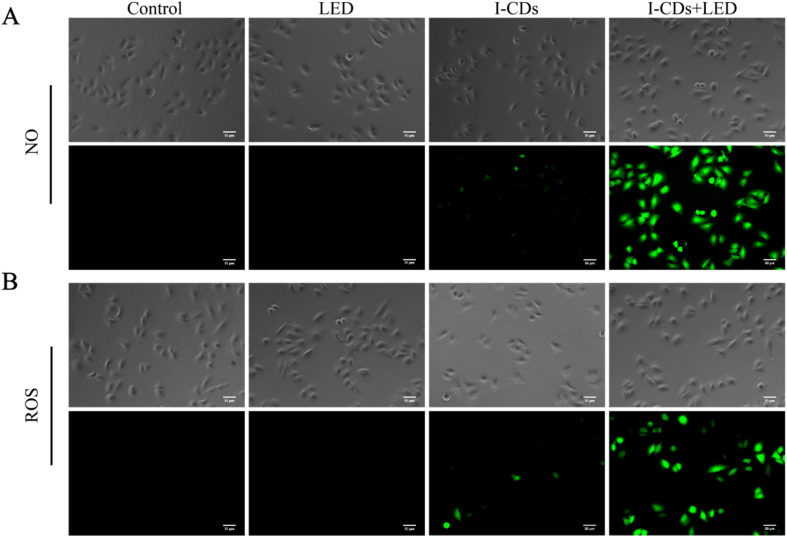
Fluorescent micrographs of A549 cells labeled with (A) DAF-FM DA and (B) DCFH-DA after 4 hours of exposure to I-CDs (600 µg mL^−1^), followed by 10 minutes of LED illumination (5 W cm^−2^, 400–800 nm). The scale bar is 50 µm.

The generation of NO and ROS during the process of PDT was further investigated by the corresponding probes. Intracellular NO was detected with the DAF-FM DA test kit in A549 and HeLa cells treated using I-CDs.^24,57^ No obvious fluorescence was observed in A549 and HeLa cells stained with pure DAF-FM DA and DCFH-DA (Fig. S5). As depicted in Fig. 5A, after irradiation with an LED light (5 W cm^−2^, 400–800 nm) for 10 minutes, the I-CD-treated A549 cells displayed intense green fluorescence under fluorescence microscopy, indicating the significant accumulation of NO in the tumor cells after incubation with I-CDs, followed by LED illumination.^58,59^ Subsequently, DCFH-DA fluorescent probes were employed to examine the production of ROS in A549 cells under identical incubation and irradiation conditions as described above.^60^ As shown in Fig. 5B, a notable increase in ROS generation was observed in the I-CD-treated A549 cells, followed by light irradiation.^61^ To verify the universality and reproducibility of the above results, the same experiments were performed in HeLa cells under identical conditions. As shown in Fig. S6, HeLa cells also exhibited significantly enhanced intracellular NO and ROS fluorescence signals upon combined treatment with I-CDs and light, with a trend consistent with that observed for A549 cells. These results confirmed that I-CDs can individually promote NO and ROS generation in different tumor cells under LED irradiation, supporting their potential as nanophotosensitizers for photodynamic therapy.^62^

### 3.6. Cellular phototoxicity of I-CDs

Based on the above experimental results verifying that I-CDs can efficiently induce the generation of intracellular ROS and NO under light excitation, we hypothesized that I-CDs could cause the damage or death of tumor cells *via* photodynamic therapy. To evaluate the PDT efficacy of I-CDs against tumor cells, the calcein AM/PI cell viability and cytotoxicity assay kit was employed, in which live cells were stained by calcein-AM and exhibited green fluorescence, while dead cells were stained by propidium iodide (PI) and exhibited red fluorescence.

**Fig. 6:**
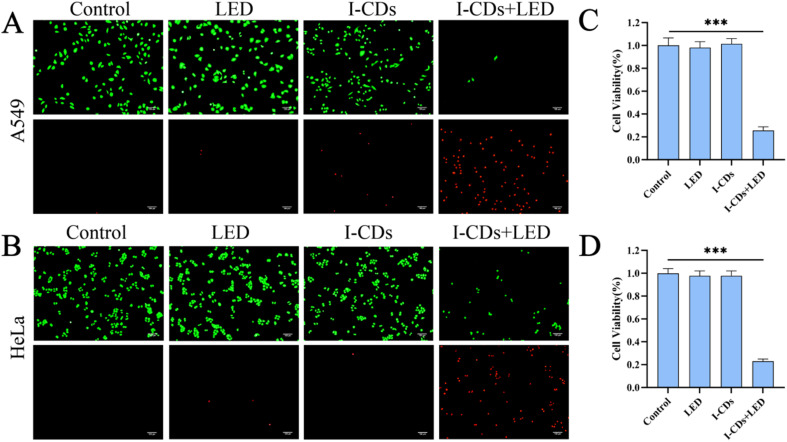
(A) Fluorescence images of A549 cells stained using calcein-AM/PI after 4 hours of exposure to I-CDs (600 µg mL^−1^), followed by 10 minutes of LED illumination (5 W cm^−2^, 400–800 nm). (B) Fluorescence images of HeLa cells stained using calcein-AM/PI after 4 hours of exposure to I-CDs (600 µg mL^−1^), followed by 10 minutes of LED illumination (5 W cm^−2^, 400–800 nm). (C) Survival of A549 cells exposed to I-CDs (600 µg mL^−1^) for 4 hours, followed by 10 minutes of LED illumination (5 W cm^−2^, 400–800 nm). (D) Survival of HeLa cells exposed to I-CDs (600 µg mL^−1^) for 4 hours, followed by 10 minutes of LED illumination (5 W cm^−2^, 400–800 nm). The scale bar is 100 µm.

As shown in Fig. 6A (A549 cells) and Fig. 6B (HeLa cells), tumor cells in the control group under dark conditions and the LED-only irradiation group displayed uniform green fluorescence with no obvious red fluorescence signals, indicating that the cells maintained good viability. Similarly, almost no red fluorescence was observed in the group treated with I-CDs alone without light stimulation, suggesting that I-CDs themselves exhibited no obvious cytotoxicity in the absence of light. In contrast, a small fraction of I-CDs treated A549 cells died after 2 minutes of LED irradiation, with a marked increase in the number of dead cells observed at 6 minutes (Fig. S7). Cells treated with I-CDs and irradiated with LED light for 10 minutes showed extensive red fluorescence accompanied by scattered green fluorescence in both cell lines, indicating a sharp increase in the proportion of dead cells and confirming that I-CDs exerted a potent killing effect on tumor cells under light excitation.^58,59^

To further quantify the killing effect and verify the results of the calcein-AM/PI assay, the MTT assay was performed to determine cell viability. Neither LED irradiation alone nor I-CD treatment alone significantly affected the viability of the two tumor cell lines. However, the cell viabilities of A549 (Fig. 6C) and HeLa cells (Fig. 6D) in the I-CD + LED group decreased to 22.9% and 25.6%, respectively, which is significantly lower than those in the control and LED-only groups (*P* < 0.001). These data clearly demonstrate that I-CDs possess favorable biocompatibility as well as an outstanding photodynamic killing effect toward tumor cells upon activation by light at a specific wavelength, resulting in massive cell death. Collectively, I-CDs have great potential as a highly efficient nanophotosensitizer for tumor photodynamic therapy.

### 3.7. CT imaging capability of I-CDs *in vitro* and *in vivo*

**Fig. 7:**
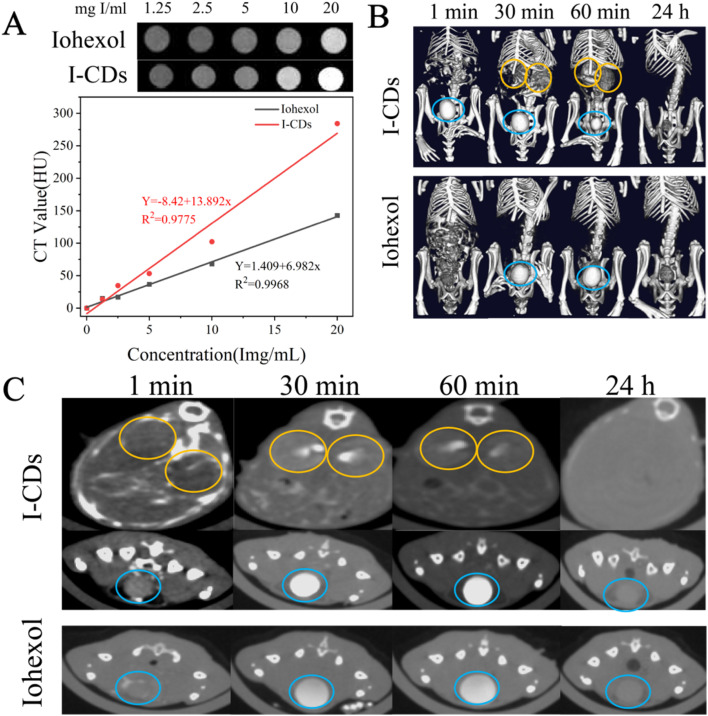
(A) CT images of iohexol and I-CDs in a PBS solution at various concentrations. (B) 3D-rendered CT images and (C) cross-sectional CT images of BALB/c mice scanned 1, 30, and 60 minutes and 24 hours after the injection of iohexol and I-CDs at a dose of 9000 µg I per mL (the yellow and blue circles represent the location of the kidneys and bladder, respectively).

Subsequently, we evaluated the X-ray attenuation performance of I-CDs using a micro-CT phantom *in vitro*. The lightness of CT scan photos of both I-CDs and iohexol augmented gradually with the enhancing iodine doses (upper panel, Fig. 7A). Moreover, the CT values of both I-CDs and iohexol increased linearly with increasing iodine doses (lower panel, Fig. 7A). The slope of the linear fit for I-CDs (13.892 HU L g^−1^) exceeded that for iohexol (6.982 HU L g^−1^). The detailed CT values of both I-CDs and iohexol are shown in Table S1. It was evident that the CT value of I-CDs exceeded that of iohexol at doses ranging from 2500 µg I per mL to 20 000 µg I per mL (Fig. 7A and Table S1, SI), confirming the superior X-ray attenuation performance of I-CDs. The *in vivo* CT imaging capability of I-CDs was further evaluated on female BALB/c mice (6 weeks old).^38^ PBS, iohexol, and I-CD solutions were intraperitoneally injected into mice, individually. It is worth noting that the bladder and kidneys of the mice subjected to PBS were hardly visible within 1 minute post-injection (Fig. S8, SI). As demonstrated in Fig. 7B (3D images) and Fig. 7C (cross-sectional images), after the intraperitoneal administration of I-CDs and iohexol at the same iodine concentration (9000 µg I per mL in PBS solution), I-CDs rapidly reached the bladder and kidneys of mice within 1 minute post-injection. The high-density contrast enhancement signals were primarily in the kidneys (yellow circle) and bladder (blue circle) and remained clearly visible after 60 minutes (upper two panels, Fig. 7C). Following iohexol administration, pronounced high-density opacities were confined to the bladder, whereas the renal parenchyma was largely devoid of contrast-related hyperdensity (lower panel, Fig. 7C). Furthermore, the intensity of signals of I-CDs in the bladder was superior to that of iohexol within 60 minutes. Similar to iohexol, the CT signals of I-CDs in the bladder were almost invisible after 24 hours, indicating the efficient renal clearance of I-CDs (Fig. 7C). The cross-sectional CT values corresponding to the kidneys and bladders of mice treated with I-CDs and iohexol were further measured using ImageJ.^63^ Notably, in the absence of contrast agent leakage, no significant signal enhancement was observed in the kidneys of mice injected with iohexol. This may be attributed to the fact that the peak enhancement of the renal cortex typically occurs 30–60 seconds after injection, and the scanning time point in this study completely missed the transient enhancement peak of iohexol.^21,64–66^ In sharp contrast, at the same iodine dosage, I-CDs exhibited longer retention properties than iohexol, which avoids the “time window” problem of the transient signal peak. Subsequently, the cross-sectional CT values of the kidneys and bladder were quantified using ImageJ software (Table S2), further confirming that the imaging performance of I-CDs is superior to that of iohexol. The comparison results reconfirmed that the imaging performance of I-CDs for the kidneys and bladder of mice was superior to that of iohexol (Table S2, SI). The aforementioned outcomes suggest that I-CDs may be a desirable CT contrast reagent for urography-related studies.

### 3.8. *In vivo* toxicity assessment of I-CDs

**Fig. 8:**
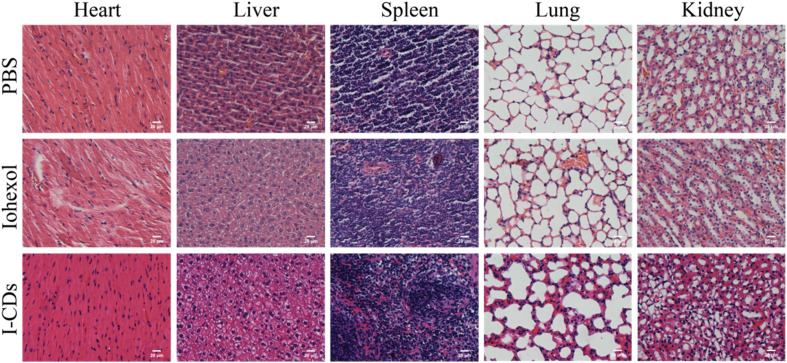
H&E staining images of the major organs (the heart, liver, spleen, lungs, and kidneys) of mice after intraperitoneal administration with PBS, iohexol and I-CDs for 48 hours. The scale bar is 20 µm.

To evaluate the *in vivo* toxicity of I-CDs, histopathological analysis was performed on the experimental animals after completing *in vivo* CT imaging. At 48 hours post-intraperitoneal injection of PBS, iohexol, and I-CDs, the mice in the three groups were anaesthetized with isoflurane, and their major organs (heart, liver, spleen, lungs, and kidneys) were collected for H&E staining. Compared with the PBS group, the I-CD group showed no obvious morphological or pathological alterations in major organs under light microscopy. Specifically, the heart exhibited orderly arranged myocardial fibers without necrosis, inflammatory cell infiltration, or interstitial edema. The liver displayed intact hepatic lobules and regularly arranged hepatic cell cords, with no steatosis, necrosis, or inflammatory infiltration. The spleen presented a clear boundary between the white pulp and red pulp, with a normal lymphocyte distribution and no abnormal hyperplasia or atrophy. The lungs showed intact alveolar structures without congestion, edema, or inflammatory cell infiltration. Finally, the kidneys exhibited clear glomerular and tubular structures, with no cast formation, epithelial necrosis, or interstitial inflammation.^20^ These results demonstrate that I-CDs do not induce acute tissue damage or inflammatory responses in mice at the experimental dose, showing extremely low *in vivo* toxicity and excellent biocompatibility (Fig. 8).

## 4. Conclusion

In summary, I-CDs were fabricated from iohexol and arginine *via* a one-pot hydrothermal method and employed as a fluorescence/CT dual-modal imaging probe. Compared with conventional iodine-based contrast agents, I-CDs achieved higher Hounsfield unit (HU) values at equivalent doses of iodine. Moreover, the fast CT imaging capacity of I-CDs for the kidneys and bladder and the efficient clearance of I-CDs through the kidneys made them suitable for urinary systemic CT contrast imaging. Furthermore, under LED irradiation, I-CDs were an effective nanophotosensitizer for the photodynamic therapy (PDT) of A549 and HeLa cells. I-CDs showed potential in the fields of fluorescence/CT bimodal imaging and PDT.

## Author contributions

Binghan Guan carried out most of the experiments and wrote the original manuscript; Jin Li acquired funding; Tingting Liang assisted in data curation; Yaoyao Zhang and Hanqin Wang helped in the explaining of data; Bifu Hu supervised this work; and Xiaobo Wang acquired funding and revised the manuscript for publication.

## Conflicts of interest

There are no conflicts to declare.

## Supplementary Material

RA-016-D6RA00126B-s001

## Data Availability

The data supporting this article have been included in the manuscript and as part of the supplementary information (SI). Supplementary information is available. See DOI: https://doi.org/10.1039/d6ra00126b.

## References

[cit1] Augustine R., Mamun A. A., Hasan A., Salam S. A., Chandrasekaran R., Ahmed R., Thakor A. S. (2021). Imaging cancer cells with nanostructures: prospects of nanotechnology driven non-invasive cancer diagnosis. Adv. Colloid Interface Sci..

[cit2] Jiang Z., Zhang M., Li P., Wang Y., Fu Q. (2023). Nanomaterial-based CT contrast agents and their applications in image-guided therapy. Theranostics.

[cit3] Huang Y., He S., Cao W., Cai K., Liang X.-J. (2012). Biomedical nanomaterials for imaging-guided cancer therapy. Nanoscale.

[cit4] Caschera L., Lazzara A., Piergallini L., Ricci D., Tuscano B., Vanzulli A. (2016). Contrast agents in diagnostic imaging: present and future. Pharmacol. Res..

[cit5] Lee N., Choi S. H., Hyeon T. (2013). Nano-Sized CT Contrast Agents. Adv. Mater..

[cit6] Torres M. J., Trautmann A., Böhm I., Scherer K., Barbaud A., Bavbek S., Bonadonna P., Cernadas J. R., Chiriac A. M., Gaeta F. (2021). *et al.*, Practice parameters for diagnosing and managing iodinated contrast media hypersensitivity. Allergy.

[cit7] Ke H., Yue X., Wang J., Xing S., Zhang Q., Dai Z., Tian J., Wang S., Jin Y. (2014). Gold Nanoshelled Liquid Perfluorocarbon Nanocapsules for Combined Dual Modal Ultrasound/CT Imaging and Photothermal Therapy of Cancer. Small.

[cit8] Xu X., Chong Y., Liu X., Fu H., Yu C., Huang J., Zhang Z. (2019). Multifunctional nanotheranostic gold nanocages for photoacoustic imaging guided radio/photodynamic/photothermal synergistic therapy. Acta Biomater..

[cit9] Wang M., Chang M., Chen Q., Wang D., Li C., Hou Z., Lin J., Jin D., Xing B. (2020). Au2Pt-PEG-Ce6 nanoformulation with dual nanozyme activities for synergistic chemodynamic therapy/phototherapy. Biomaterials.

[cit10] Wang S., Li X., Chen Y., Cai X., Yao H., Gao W., Zheng Y., An X., Shi J., Chen H. (2015). A Facile One-Pot Synthesis of a Two-Dimensional MoS2/Bi2S3 Composite Theranostic Nanosystem for Multi-Modality Tumor Imaging and Therapy. Adv. Mater..

[cit11] Badrigilan S., Shaabani B., Gharehaghaji N., Mesbahi A. (2019). Iron oxide/bismuth oxide nanocomposites coated by graphene quantum dots: “Three-in-one” theranostic agents for simultaneous CT/MR imaging-guided in vitro photothermal therapy. Photodiagnosis Photodyn. Ther..

[cit12] Le W., Cui S., Chen X., Zhu H., Chen B., Cui Z. (2016). Facile Synthesis of Gd-Functionalized Gold Nanoclusters as Potential MRI/CT Contrast Agents. Nanomaterials.

[cit13] Wang M., Su Y., Liu Y., Liang Y., Wu S., Zhou N., Shen J. (2022). Antibacterial fluorescent nano-sized lanthanum-doped carbon quantum dot embedded polyvinyl alcohol for accelerated wound healing. J. Colloid Interface Sci..

[cit14] Sobhanan J., Rival J. V., Anas A., Sidharth Shibu E., Takano Y., Biju V. (2023). Luminescent quantum dots: synthesis, optical properties, bioimaging and toxicity. Adv. Drug Deliv. Rev..

[cit15] Li J., Gong X. (2022). The Emerging Development of Multicolor Carbon Dots. Small.

[cit16] Wu B., Lu S.-T., Yu H., Liao R.-F., Li H., Lucie Zafitatsimo B. V., Li Y.-S., Zhang Y., Zhu X.-L., Liu H.-G. (2018). *et al.*, Gadolinium-chelate functionalized bismuth nanotheranostic agent for in vivo MRI/CT/PAI imaging-guided photothermal cancer therapy. Biomaterials.

[cit17] Molkenova A., Serik L., Ramazanova A., Zhumanova K., Duisenbayeva B., Zhussupbekova A., Zhussupbekov K., Shvets I. V., Kim K. S., Han D.-W. (2023). *et al.*, Terbium-doped carbon dots (Tb-CDs) as a novel contrast agent for efficient X-ray attenuation. RSC Adv..

[cit18] Su Y., Liu S., Guan Y., Xie Z., Zheng M., Jing X. (2020). Renal clearable Hafnium-doped carbon dots for CT/Fluorescence imaging of orthotopic liver cancer. Biomaterials.

[cit19] Wang X., Lu Y., Hua K., Yang D., Yang Y. (2021). Iodine-doped carbon dots with inherent peroxidase catalytic activity for photocatalytic antibacterial and wound disinfection. Anal. Bioanal. Chem..

[cit20] Cui Y., Yang D., Li Q., Peng Z., Zhong Z., Song Y., Han Q., Yang Y. (2024). Cu,Zn,I-Doped Carbon Dots with Boosted Triple Antioxidant Nanozyme Activity for Treatment of DSS-Induced Colitis. ACS Appl. Mater. Interfaces.

[cit21] Du F., Zhang M., Ju H., Zhang L., Sun M., Zhou Z., Dai Z., Zhang L., Gong A., Wu C. (2015). Engineering iodine-doped carbon dots as dual-modal probes for fluorescence and X-ray CT imaging. Int. J. Nanomed..

[cit22] Cui M., Tang Z., Ahmad Z., Pan C., Lu Y., Ali K., Huang S., Lin X., Wahab A., Iqbal M. Z. (2024). *et al.*, Facile synthesis of manganese-hafnium nanocomposites for multimodal MRI/CT imaging and in vitro photodynamic therapy of colon cancer. Colloids Surf., B.

[cit23] Bogdan C. (2015). Nitric oxide synthase in innate and adaptive immunity: an update. Trends Immunol..

[cit24] Wu X., Wang M., Yu F., Cai H., Tedesco A. C., Li Z., Bi H. (2024). Core–shell structured carbon dots with up-conversion fluorescence and photo-triggered nitric oxide-releasing properties. Analyst.

[cit25] Liu X., Liu Y., Thakor A. S., Kevadiya B. D., Cheng J., Chen M., Li Y., Xu Q., Wu Q., Wu Y. (2021). *et al.*, Endogenous NO-releasing Carbon Nanodots for Tumor-specific Gas Therapy. Acta Biomater..

[cit26] Fang X., Cai S., Wang M., Chen Z., Lu C., Yang H. (2021). Photogenerated Holes Mediated Nitric Oxide Production for Hypoxic Tumor Treatment. Angew. Chem., Int. Ed..

[cit27] Alzeibak R., Mishchenko T. A., Shilyagina N. Y., Balalaeva I. V., Vedunova M. V., Krysko D. V. (2021). Targeting immunogenic cancer cell death by photodynamic therapy: past, present and future. J. Immunother..

[cit28] Li W., Yang J., Luo L., Jiang M., Qin B., Yin H., Zhu C., Yuan X., Zhang J., Luo Z. (2019). *et al.*, Targeting photodynamic and photothermal therapy to the endoplasmic reticulum enhances immunogenic cancer cell death. Nat. Commun..

[cit29] Wang Z., Jin A., Yang Z., Huang W. (2023). Advanced Nitric Oxide Generating Nanomedicine for Therapeutic Applications. ACS Nano.

[cit30] Huang D., Jing G., Zhang L., Chen C., Zhu S. (2021). Interplay Among Hydrogen Sulfide, Nitric Oxide, Reactive Oxygen Species, and Mitochondrial DNA Oxidative Damage. Front. Plant Sci..

[cit31] Han C., Yu Q., Jiang J., Zhang X., Wang F., Jiang M., Yu R., Deng T., Yu C. (2021). Bioenzyme-responsivel-arginine-based carbon dots: the replenishment of nitric oxide for nonpharmaceutical therapy. Biomater. Sci..

[cit32] Feng L., He F., Dai Y., Gai S., Zhong C., Li C., Yang P. (2017). Multifunctional UCNPs@MnSiO3@g-C3N4nanoplatform: improved ROS generation and reduced glutathione levels for highly efficient photodynamic therapy. Biomater. Sci..

[cit33] Kudoand S., Nagasaki Y. (2015). A novel nitric oxide-based anticancer therapeutics by macrophage-targeted poly(l-arginine)-based nanoparticles. J. Contr. Release.

[cit34] Bader J. E., Voss K., Rathmell J. C. (2020). Targeting Metabolism to Improve the Tumor Microenvironment for Cancer Immunotherapy. Mol. Cell.

[cit35] Su H., Liao Y., Wu F., Sun X., Liu H., Wang K., Zhu X. (2018). Cetuximab-conjugated iodine doped carbon dots as a dual fluorescent/CT probe for targeted imaging of lung cancer cells. Colloids Surf., B.

[cit36] Li J., Feng X., Jiang H., Mo X., Liu C., Liu X., Feng T., Zhou Y. (2025). Near-infrared and pH-responsive carbon dots/bergenin for biological imaging and chemo-photothermal synergistic tumor therapy. J. Colloid Interface Sci..

[cit37] Kuan C.-H., Zhang X., Krueger T. D., Lancaster L. S., Chen C., Yeasmin S., Ullah A., Cheng L.-J., Fang C. (2025). Dissecting the Large Stokes Shift Fluorescence of Graphene-Sheet-Based Carbon Dots by Excitation-Dependent Ultrafast Spectroscopy. J. Phys. Chem. Lett..

[cit38] Jeong Y., Jin M., Kim K. S., Na K. (2022). Biocompatible carbonized iodine-doped dots for contrast-enhanced CT imaging. Biomater. Res..

[cit39] Szapoczka W. K., Truskewycz A. L., Skodvin T., Holst B., Thomas P. J. (2023). Fluorescence intensity and fluorescence lifetime measurements of various carbon dots as a function of pH. Sci. Rep..

[cit40] Mandal S., Prasad S. R., Mandal D., Das P. (2019). Bovine Serum Albumin Amplified Reactive Oxygen Species Generation from Anthrarufin-Derived Carbon Dot and Concomitant Nanoassembly for Combination Antibiotic–Photodynamic Therapy Application. ACS Appl. Mater. Interfaces.

[cit41] Yue W., Fang Z., Yu T., Wang W., Yu H., Wu Z., Li X., Yangzom G., Lu X., Wu Q. (2025). *et al.*, An Arginine-Inspired Nanocomposite Enhances Tumor Oxygenation for Optimized Photodynamic Therapy. ACS Appl. Mater. Interfaces.

[cit42] Antoine C., Sahylí O. P. M., Ricci-Junior E., Magalhães R. A. L., Santos-Oliveira R. (2022). Graphene quantum dots as bimodal imaging agent for X-ray and Computed Tomography. Eur. J. Pharm. Biopharm..

[cit43] Wang L., Wu J., Wang B., Xing G., Qu S. (2025). d-arginine-functionalized carbon dots with enhanced near-infrared emission and prolonged metabolism time for tumor fluorescent-guided photothermal therapy. J. Colloid Interface Sci..

[cit44] Wei S., Shi X., Wang C., Zhang H., Jiang C., Sun G., Jiang C. (2023). Facile synthesis of nitrogen-doped carbon dots as sensitive fluorescence probes for selective recognition of cinnamaldehyde and l-arginine/l-lysine in living cells. Spectrochim. Acta, Part A.

[cit45] Ristić B., Trpkov Đ., Dojčilović R., Đukić T., Božanić D. K., Stefanović R., Vasić B., Drvenica I. (2025). Nitrogen-doped carbon dots as biocompatible fluorescent agents for labelling human red blood cells. Biomater. Adv..

[cit46] Yang H., Liu Y., Guo Z., Lei B., Zhuang J., Zhang X., Liu Z., Hu C. (2019). Hydrophobic carbon dots with blue dispersed emission and red aggregation-induced emission. Nat. Commun..

[cit47] Zhao M., Lin M., Guo G., Xia Y. (2024). Polarity-Targeted Carbon Dots for Mitochondria and Lysosomes Imaging. Anal. Chem..

[cit48] Atchudan R., Edison T. N. J. I., Lee Y. R. (2016). Nitrogen-doped carbon dots originating from unripe peach for fluorescent bioimaging and electrocatalytic oxygen reduction reaction. J. Colloid Interface Sci..

[cit49] Samarehfekri H., Rahimi H. R., Ranjbar M. (2020). Controlled and cellulose eco-friendly synthesis and characterization of Bi2O2CO3 quantum dot nanostructures (QDNSs) and drug delivery study. Sci. Rep..

[cit50] Đorđević L., Arcudi F., Prato M. (2019). Preparation, functionalization and characterization of engineered carbon nanodots. Nat. Protoc..

[cit51] Pei J., Li H., Chen F., Chen Z., Yuan X., Han Z., Chen D., Yu D., Zhang D. (2024). Lanthanide Functionalized Carbon Quantum Dots for White Light Emission, pH Sensing, and Co (II) Detection. ACS Appl. Mater. Interfaces.

[cit52] Kamble P., Malavekar D., Tiwari A. P. (2023). Natural Biowaste Derived Fluorescent Carbon Quantum Dots: Synthesis, Characterization and Biocompatibility Study. J. Fluoresc..

[cit53] Li S., Guo Z., Zhang Y., Xue W., Liu Z. (2015). Blood Compatibility Evaluations of Fluorescent Carbon Dots. ACS Appl. Mater. Interfaces.

[cit54] Unnikrishnan B., Wu R.-S., Wei S.-C., Huang C.-C., Chang H.-T. (2020). Fluorescent Carbon Dots for Selective Labeling of Subcellular Organelles. ACS Omega.

[cit55] Qin H., Sun Y., Geng X., Zhao K., Meng H., Yang R., Qu L., Li Z. (2020). A wash-free lysosome targeting carbon dots for ultrafast imaging and monitoring cell apoptosis status. Anal. Chim. Acta.

[cit56] Hammoudeh S. M., Hammoudeh A. M., Hamoudi R. (2019). High-throughput quantification of the effect of DMSO on the viability of lung and breast cancer cells using an easy-to-use spectrophotometric trypan blue-based assay. Histochem. Cell Biol..

[cit57] Yao M., He Q., Tao Y., Kang X., Wu X., Shi F., Wei Y., Liu J., Meng Z., Gu R. (2025). *et al.*, Chitosan-Derived Carbon Quantum Dots with Dual ROS Scavenging and Anti-inflammatory Functionalities for Accelerated Wound Repair. ACS Appl. Mater. Interfaces.

[cit58] Wang S., McCoy C. P., Li P., Li Y., Zhao Y., Andrews G. P., Wylie M. P., Ge Y. (2024). Carbon Dots in Photodynamic/Photothermal Antimicrobial Therapy. Nanomaterials.

[cit59] Yue J., Miao P., Li L., Yan R., Dong W.-F., Mei Q. (2022). Injectable Carbon Dots-Based Hydrogel for Combined Photothermal Therapy and Photodynamic Therapy of Cancer. ACS Appl. Mater. Interfaces.

[cit60] Zhang B., Wang H., Yu L., Ma Y., Ren G., Ma S., Li L., Guo L., Xu S., Yan L. (2026). *et al.*, Cascade responsive cell membrane biomimetic nanoplatform for synergistic therapy of esophageal cancer. Colloids Surf., B.

[cit61] Fu L., Huang Y., Sun X., Wang X., Li S., Wang X., Kang Q., Shen D., Chen L. (2025). A Hyaluronidase-Responsive Nanoplatform for Near-Infrared Fluorescence Imaging and Synergistic Photodynamic/Gas/Chemodynamic Therapy in Bacterial Infection Sites. ACS Appl. Mater. Interfaces.

[cit62] Sun S., Chen J., Jiang K., Tang Z., Wang Y., Li Z., Liu C., Wu A., Lin H. (2019). Ce6-Modified Carbon Dots for Multimodal-Imaging-Guided and Single-NIR-Laser-Triggered Photothermal/Photodynamic Synergistic Cancer Therapy by Reduced Irradiation Power. ACS Appl. Mater. Interfaces.

[cit63] Xu M., Qi Y., Peng Y., Lu H., Chen Y., Wang Z., Lin Y., Jiang X., Du B. (2025). Reno-protective CT contrast nanoagent targets proximal tubular epithelium for kidney disease imaging and repair in a mouse model. Nat. Commun..

[cit64] Hooper L., Heung M., Wang L., Matvekas A., Alikhani R., Kenes M. T., Stringer K. A., Mueller B. A., Pai M. P. (2025). Pharmacokinetic Characterization of Iopamidol and Iohexol for Optimizing Measured Glomerular Filtration Rate Assessment in Clinical Practice and Drug Development. Clin. Pharmacol..

[cit65] Siow J. W., Chau J., Podadera J. M., Makara M. (2022). Investigation of scan delays for CT evaluation of inner wall layering and peak enhancement of the canine stomach and small intestine using a 20 second fixed-injection-duration and bolus tracking technique. Vet. Radiol. Ultrasound.

[cit66] Gu X., Zhu Z., Fan Q., Wei Y., Wang G., Meng F., Zhong Z., Deng C. (2019). Nanoagents Based on Poly(ethylene glycol)-b-Poly(l-thyroxine) Block Copolypeptide for Enhanced Dual-Modality Imaging and Targeted Tumor Radiotherapy. Small.

