# Supplementary material for: Iodine-doped carbon dots for fluorescence/computed tomography imaging and photodynamic therapy of tumor cells
Source: RSC Adv. 2026 Mar 30;16(19):17401–14. doi: 10.1039/d6ra00126b (PMC13034852; doi:10.1039/d6ra00126b)
Supplement: RA-016-D6RA00126B-s001 [file RA-016-D6RA00126B-s001.pdf]

Supplementary Information (SI) for RSC Advances.
This journal is © The Royal Society of Chemistry 2026

Supplementary Information

Binghan Guan[a,1], Jin Li[b,1], Tingting Liang[a], Yaoyao Zhang[a], Hanqin Wang[a], Bifu Hu[c*], Xiaobo Wang[a,b*]

[a] Center for translational medicine, Suizhou Hospital, Hubei University of Medicine, Suizhou 441300, People's Republic of China

[b] Reproductive medicine center & Gynaecology ward 2, Suizhou Hospital, Hubei University of Medicine, Suizhou 441300, People's Republic of China

[c] Department of radiology, Suizhou Hospital of Traditional Chinese Medicine, Suizhou 441300, People's Republic of China

[1] These authors contributed equally to this work

[*] Corresponding author

Emails addresses : xbwang@hbmu.edu.cn (X. Wang), 156689075@qq.com (B. Hu).

Tables, Figures.

Table S1. Comparison of CT values of Iohexol and I-CDs at the same concentration of iodine. S5

Fig. S1. Fluorescence lifetime of I-CDs. S5

Fig. S2. UV-vis spectrum of Arginine and Iohexol........ S6

Fig. S3. FTIR spectrum of Arginine, Iohexol and I-CDs. S6

Fig. S4. Serum Stability Assay of I-CDs. S7

Fig. S5. Fluorescence images of A549 and HeLa cells stained with pure (A) DAF-FM DA and (B) DCFH-DA........ S8

Fig. S6. Fluorescence images of HeLa cells stained with (A) DAF-FM DA and (B) DCFH-DA after a 4 hours exposure to I-CDs, followed by LED illumination........ S7

Fig. S7. Fluorescence pictures of A549 cells stained using Calcein-AM/PI after a 4 hours exposure to I-CDs (600 μg mL^{-1}) succeeded by 2,4 and 6 minutes LED illumination (5 W cm^{-2}, 400-800 nm)........ S9

Fig. S8. 3D-rendered CT images of *BALB/c* mice scanned at 1minute after injection of PBS S9

Table S2. Comparison of cross-sectional CT values corresponding to the kidneys and bladders of mice subjected to I-CDs and Iohexol at the same concentration of iodine..S10

Iodine-doped carbon dots for fluorescence/CT imaging and photodynamic therapy of tumor cells

1. Materials

Calcein-AM/PI, Mito-Tracker Green, Lyso-Tracker Green Hoechst 33342, Nitric oxide Detect Kit (DF-FM DA) and Reactive oxygen species Detect Kit (DCFH-DA) were purchased from Shanghai Biyun Tian Biotechnology Co., LTD. Methyl thiazolyl diphenyltetrazolium bromide (MTT) was purchased from Shanghai Biotech Biotechnology Co., LTD. Iohexol (SKU: I134719, purity: 98%) was purchased from Aladdin Industrial Company in Shanghai, China. L-Arginine (SKU: A640158, purity: 98%) was sourced from Sinopharm Chemical Reagent Co., Ltd. in Shanghai, China. Fetal bovine serum, penicillin-streptomycin and A549 cells were purchased from Procell (Wuhan, China). HeLa cells and HEK-293T were provided by Shanghai Cell Bank, Chinese Academy of Sciences (Shanghai, China). Dialysis bags (MWCO:500-1000 Da) were purchased from Shanghai Yuanye Biotechnology Co., Ltd. in China. Other analytical grade reagents were used directly without any additional treatment. Ultra-pure water was produced by the SYS-II-10L ultra-pure water machine (Chengdu Shenyuan Technology Co., LTD., China).

2. Cell toxicity and haemocompatibility test

Cell toxicity tests were performed as the following steps:

① Cell Seeding: HEK-293T, A549, and HeLa cells in good growth condition were used. For each cell type, the cells were gently washed twice with 2 mL of PBS buffer. Subsequently, 1 mL of trypsin-EDTA solution was added to each dish for cell detachment. The trypsin was then aspirated, and the digestion was terminated by adding fresh complete medium supplemented with 10% fetal bovine serum (FBS). The cell suspension was centrifuged at 2000 rpm for 3 minutes to pellet the cells in the logarithmic growth phase. After centrifugation, the pellet was resuspended in fresh medium. Cell density was determined by counting 10 μL of the diluted suspension using a hemocytometer. Based on the count, the cell density was adjusted, and the cells were seeded into a 96-well plate at a density of 1×10^4 cells per well. The peripheral wells were filled with sterile phosphate-buffered saline (PBS) to

minimize evaporation effects. The plate was then incubated overnight at 37°C in a humidified atmosphere containing 5% CO_2.

② I-CDs treatment: After overnight incubation, the old culture medium was carefully aspirated from each well. The cells were then treated with 100 μL of fresh medium containing varying concentrations of I-CDs (0, 50, 100, 200, 400, 600, 800, and 1000 μg mL^{-1}). The treatment continued for 24 hours. Each concentration was tested in six replicate wells. Two control groups were included: a blank control (medium only, without cells) and a negative control (cells treated with medium only, without I-CDs).

③ MTT Incubation and Formazan Dissolution: Following the 24 hours treatment period, the plate was retrieved from the incubator. Then, 20 μL of MTT reagent (5000 μg mL^{-1} in PBS) was added to each well, and the plate was protected from light and incubated for an additional 4 hours. After incubation, the supernatant containing the MTT solution was carefully removed. To dissolve the formed purple formazan crystals, 150 μL of dimethyl sulfoxide (DMSO) was added to each well. The plate was then placed on a thermostatic shaker at 37°C and gently agitated at low speed for 10 minutes to ensure complete dissolution. Spectrophotometric Measurement: The absorbance of the solution in each well was measured at a wavelength of 490 nm using a microplate spectrophotometer (BioTek, Epoch). Cell viability was calculated according to the following formula:

$$\text{Cell viability (\%)} = \left(\frac{A_{\text{test}} - A_{\text{blank}}}{A_{\text{control}} - A_{\text{blank}}}\right) \times 100\% \qquad (1)$$

Haemocompatibility tests were performed as the following steps::

① Red Blood Cell (RBC) Isolation: Fresh human blood, anticoagulated with heparin, was collected from the affiliated hospital. For RBC isolation, a 2 mL aliquot of blood was repeatedly washed with phosphate-buffered saline (PBS) until the supernatant became clear and colorless. The washing process involved centrifugation at 3000 rpm for 5 minutes at each step. The final RBC pellet was collected after the last centrifugation.

② Sample Preparation and Experimental Groups: The isolated RBCs were

resuspended in PBS to prepare a 2% (v/v) RBC suspension. For the experimental groups, 100 μL of this RBC suspension was mixed with an equal volume (100 μL) of PBS solutions containing I-CDs at varying final concentrations (0, 25, 50, 100, 200, 400, 600, 800 μg/mL). Two control groups were established: a positive control (100 μL RBC suspension mixed with 100 μL ultrapure water) and a negative control (100 μL RBC suspension mixed with 100 μL PBS only).

③ Incubation and Detection: All prepared sample and control mixtures were incubated in a 37℃ thermostatic shaker at 50 rpm for 2 hours. After incubation, the tubes were left undisturbed for 5 minutes to allow sedimentation. Photographs of each group were taken to visually document hemolysis. Subsequently, the supernatant from each tube was carefully transferred to a 96-well plate (with three replicate wells per sample). The absorbance of the supernatants was measured at 490 nm using a microplate reader (BioTek, Epoch). The hemolysis rate (HR) was calculated using the following formula:

$$\mathrm{HR}(\%) = \left(\frac{OD_{\text{sample}} - OD_{\text{negative}}}{OD_{\text{positive}} - OD_{\text{negative}}} \right) \times 100\% \tag{2}$$

mg I/mL	**0**	**1.25**	**2.5**	**5**	**10**	**20**
Iohexol(HU)	0	15.1	16.9	36.5	67.9	142.6
I-CDs(HU)	0	13.7	34.6	53.2	102	284.3

Table S1. Comparison of CT values of Iohexol and I-CDs at the same concentration of iodine. The unit of CT value was Hounsfield Unit (HU).

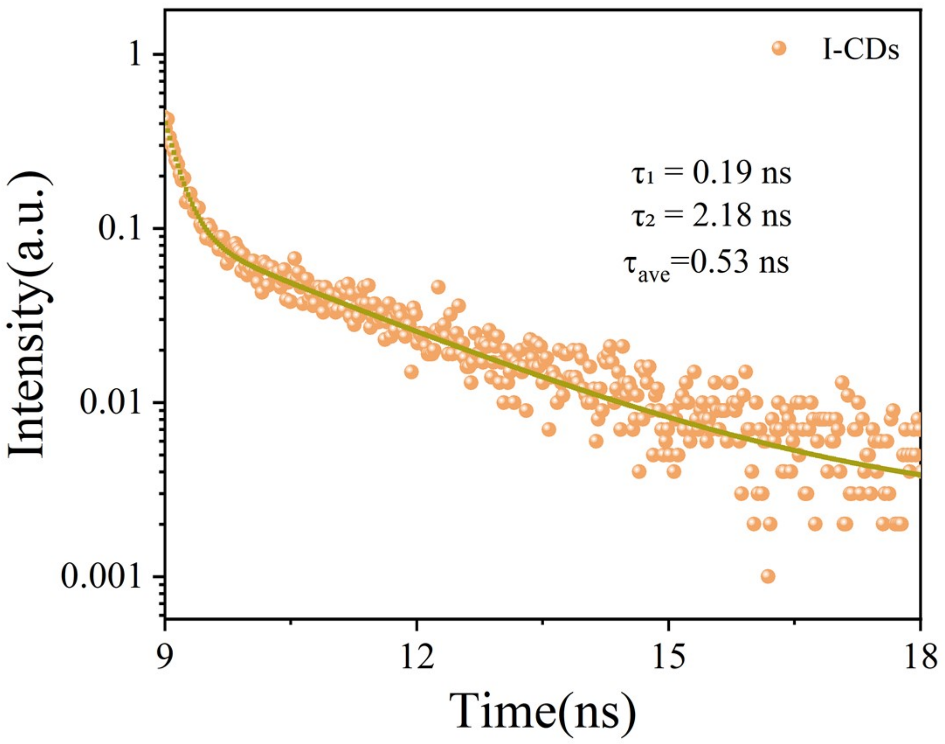

Fig. S1. Fluorescence lifetime of I-CDs.

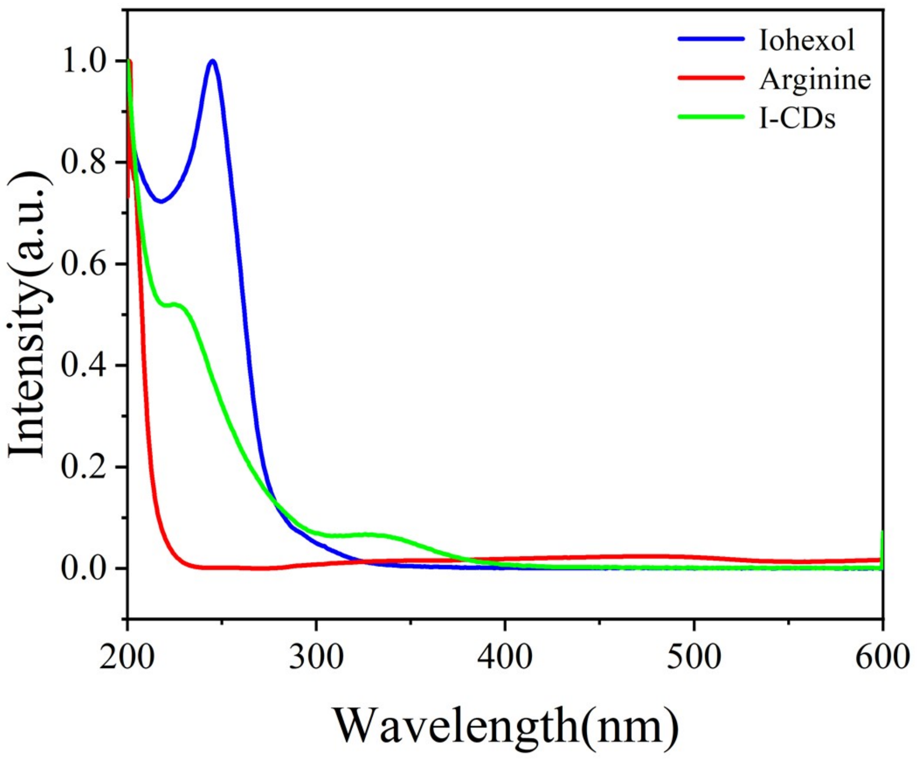

Fig. S2. UV-vis spectrum of Arginine, Iohexol, and I-CDs.

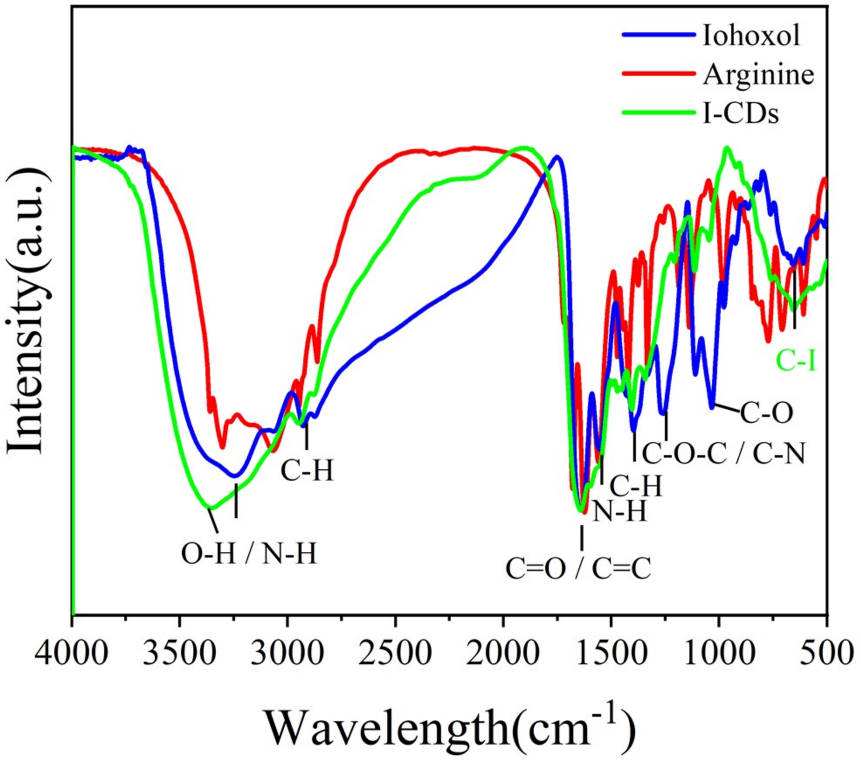

Fig. S3. FTIR spectrum of Arginine and Iohexol.

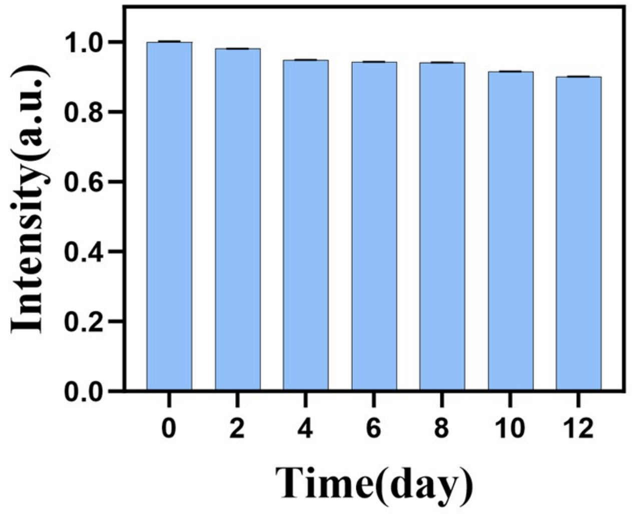

Fig. S4. Serum Stability Assay of I-CDs.

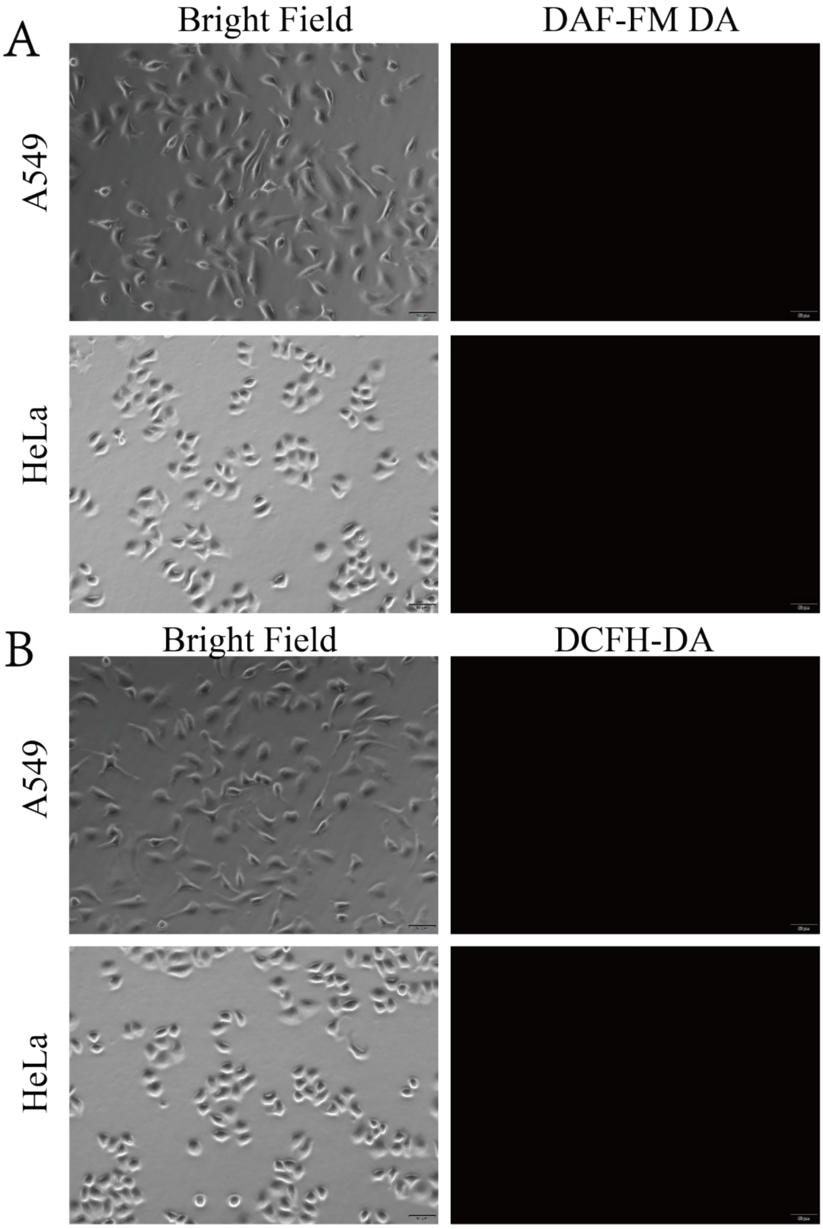

Fig. S5. Fluorescence images of A549 and HeLa cells stained with (A) DAF-FM DA and (B) DCFH-DA.

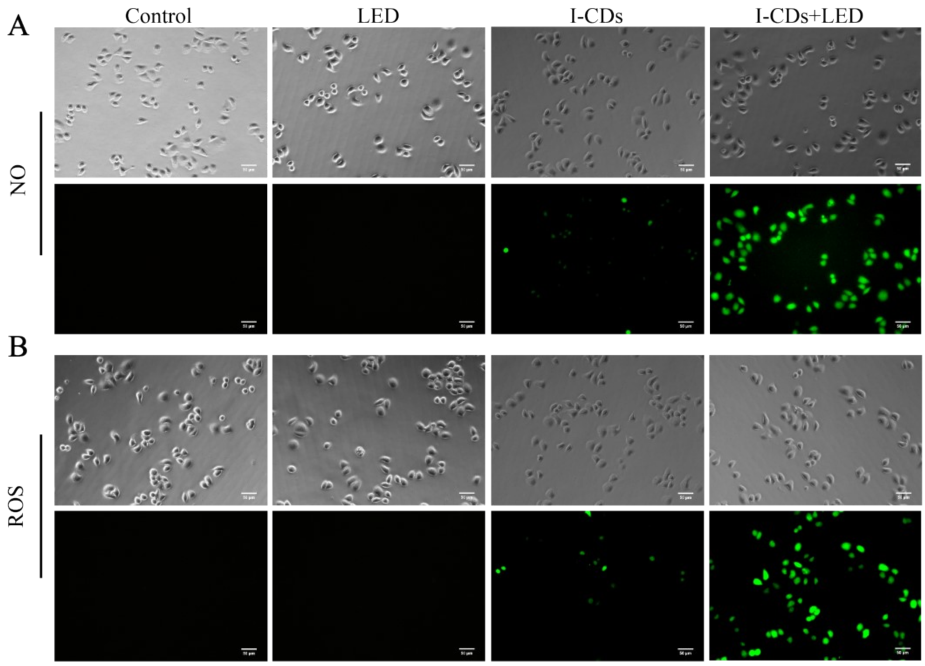

Fig. S6. Fluorescence images of HeLa cells stained with (A) DAF-FM DA and (B) DCFH-DA after a 4h exposure to I-CDs (600 μg mL^{-1}), followed by 10 minutes LED illumination (5 W cm^{-2} , 400-800 nm). Scale bar denoted 50 μm.

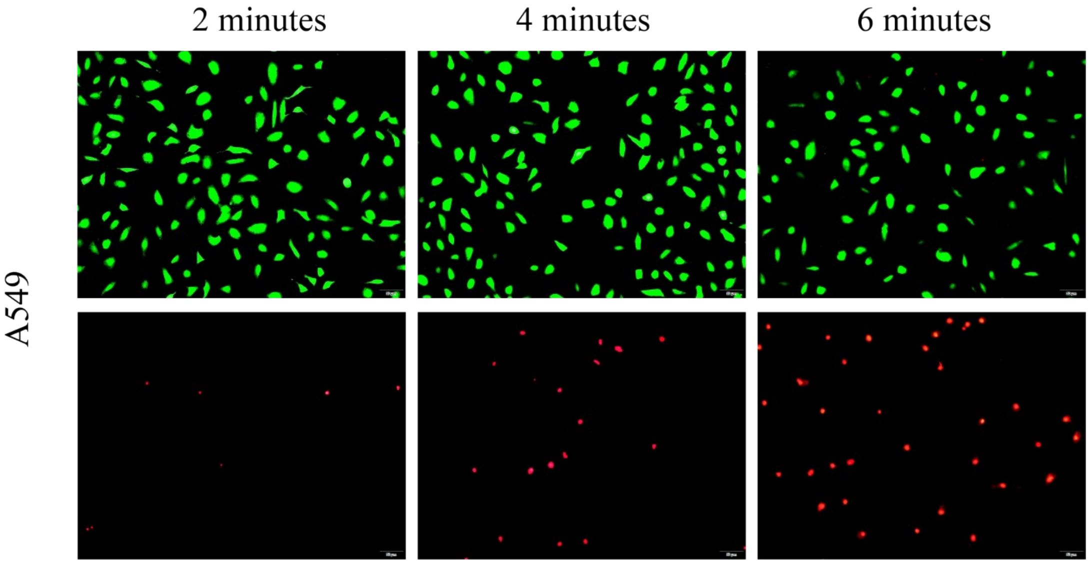

Fig. S7. Fluorescence pictures of A549 cells stained using Calcein-AM/PI after a 4 hours exposure to I-CDs (600 μg mL^{-1}) succeeded by 2 minutes, 4 minutes, and 6 minutes LED illumination (5 W cm^{-2}, 400-800 nm).

PBS

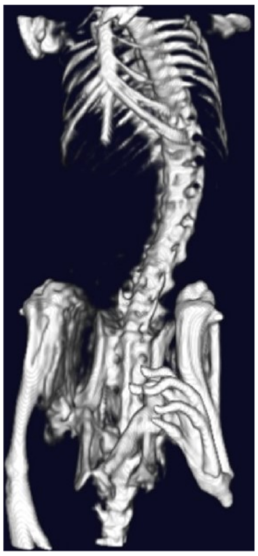

Fig. S8. 3D-rendered CT images of *BALB/c* mice scanned at 1minute after intraperitoneal injection of PBS solution.

Organ	Time	I-CDs(HU)	Iohexol(HU)
bladder	1 min	121.149	120.957
	30 min	230	167.708
	60 min	230	199.896
kidney	1 min	88.056	/
	30 min	187.641	/
	60 min	160.994	/

Table S2. Comparison of cross-sectional CT values corresponding to the kidneys and bladders of mice subjected to I-CDs and Iohexol at the same concentration of iodine. "/" denotes "No significant difference was observed in CT values between muscle tissue, kidney and bladder."
